# What are the perceived needs of people living with chronic pain regarding physiotherapy services? A scoping review protocol

**DOI:** 10.1371/journal.pone.0274730

**Published:** 2023-02-02

**Authors:** Jonathan Gervais-Hupé, Arthur Filleul, Kadija Perreault, Isabelle Gaboury, Timothy H. Wideman, Céline Charbonneau, Fatiha Loukili, Martine Gagnon, Anne Hudon

**Affiliations:** 1 School of Rehabilitation, Faculty of Medicine, Université de Montréal, Montréal, Quebec, Canada; 2 Centre for Interdisciplinary Research in Rehabilitation of Greater Montreal (CRIR) de l’Institut Universitaire sur la Réadaptation en Déficience Physique de Montréal (IURDPM), Centre Intégré Universitaire de Santé et de Services Sociaux du Centre-Sud-de-l’Ile-de-Montréal (CCSMTL), Montréal, Quebec, Canada; 3 Centre de Recherche en Ethique (CRÉ), Montréal, Quebec, Canada; 4 Université Grenoble Alpes, Saint-Martin-d’Hères, France; 5 Center for Interdisciplinary Research in Rehabilitation and Social Integration (CIRRIS), Centre Intégré Universitaire de Santé et de Services Sociaux de la Capitale-Nationale, Quebec City, Quebec, Canada; 6 Department of Rehabilitation, Faculty of Medicine, Université Laval, Quebec City, Quebec, Canada; 7 Department of Family Medicine and Emergency Medicine, Faculty of Medicine and Health Sciences, University of Sherbrooke, Sherbrooke, Quebec, Canada; 8 School of Physical and Occupational Therapy, Faculty of Medicine, McGill University, Montreal, Quebec, Canada; 9 Centre for Interdisciplinary Research in Rehabilitation of Greater Montreal (CRIR) de l’Institut Universitaire sur la Réadaptation en Déficience Physique de Montréal (IURDPM), Centre Intégré Universitaire de Santé et de Services Sociaux du Centre-Ouest-de-l’Ile-de-Montréal, Montreal, Quebec, Canada; 10 Association Québécoise de la Douleur Chronique, Montreal, Quebec, Canada; 11 Association des Personnes Vivant Avec de la Douleur Chronique, Gatineau, Quebec, Canada; 12 Bibliothèque de l’Université Laval, Quebec City, Quebec, Canada; Mugla Sitki Kocman Universitesi, TURKEY

## Abstract

**Introduction:**

Chronic pain represents a major health issue, affecting the physical and mental health of approximately one in five people worldwide. It is now widely recognized that health professionals should use interventions that meet the needs of people living with chronic pain. Therefore, physiotherapists should attend to patients’ perceived needs regarding physiotherapy services, i.e. the needs that are perceived by patients themselves based on their beliefs, values, preferences and expectations. However, previous reviews have mainly focused on health professionals’ and experts’ evaluations of patients’ needs, which may result in inadequate answers to these needs. Therefore, a better understanding of patients’ perceived needs could lead to more ethical and higher quality physiotherapy services.

**Objective:**

The aim of this scoping review is thus to explore what is known from the existing literature about the perceived needs of people living with chronic pain regarding physiotherapy services.

**Methods:**

This scoping review will follow Arksey and O’Malley’s six-step framework. Medline, Embase, CINHAL, and APA PsycINFO will be used to search the scientific literature. The grey literature will also be searched using Google Scholar, OpenGrey and ProQuest Dissertation & Theses Global (PQDTGlobal). Studies published in English and French will only be considered. Two independent reviewers will perform the selection and extraction processes. Descriptive statistics will be performed to characterize the included studies. Quantitative, qualitative and mixed methods studies will be analyzed and synthetized using convergent qualitative meta-integration. Thereby, we will use the seven steps for convergent qualitative meta-integration proposed by Frantzen and Fetters to transform, analyze and integrate the quantitative and qualitative data.

**Inclusion criteria:**

Included studies will describe the perceived needs of adults living with chronic pain regarding physiotherapy services. Studies focusing on the perspectives of health professionals and rehabilitation services other than physiotherapy will be excluded.

## Introduction

Chronic pain represents a major health issue, affecting approximately one in five persons worldwide [1]. It significantly and negatively impacts the physical and mental health of those affected [2]. Many institutions such as the Institute for Health and Care Excellence (NICE) [3], the Canadian Pain Task Force [4] and the US Department of Health and Human Services (HHS) [5] recommend that ideal care models should be patient-centered and thus answer patients’ needs. Patient-centered care is defined as a patient-therapist collaborative way of caring “that is respectful of, and responsive to, individual patient preferences, needs and values” [6].

Although patients’ needs are a key element of high-quality and patient-centered care, the term ‘needs’ remains a complex and poorly defined concept [7–9]. Two main categories of needs have been delineated, depending on who evaluates these needs: needs evaluated by experts and healthcare professionals, and those perceived by the patients themselves [8, 10]. As highlighted in a recent scoping review, the majority of studies looking at needs relating to rehabilitation services have targeted needs evaluated by services representatives or experts [11]. Using such a perspective results in a top-down approach of assessing needs and can often lead to a false understanding of the needs felt by the patients [12]. For example, in a study looking at patient-therapist interaction in musculoskeletal physiotherapy settings, patients raised a need for more patient education during the interaction with their physiotherapist while most physiotherapists questioned in the study did not mention the importance of patient education [13]. This top-down approach can also result in inappropriate health resources allocation from publicly funded health care systems. These allocations are highly dependent of needs’ indicators that rarely consider the patients’ perceived needs [14]. While patient-centered pratice has been promoted since the seventies [15], clinicians are still more likely to provide care based on their preferences rather than on their patients’ needs and preferences [16]. This lack of consideration for patients’ perceived needs could lead to poor clinical outcomes and low satisfaction [17]. It also raises ethical concerns related to patients’ autonomy and self-determination [17]. Conversely, considering patients’ perceived needs and therefore using a patient-centered approach not only allows more compassionate and empathetic care but also results in higher care satisfaction, greater quality of care and increased patients’ quality of life and wellbeing [18]. Thus, to respect and respond to patients’ needs, clinicians should consider and act upon patients’ perceived needs, i.e., needs that are perceived or felt by patients based on their beliefs, values, preferences, and expectations [8, 9, 19].

Among the healthcare services offered to people living with chronic pain, physiotherapy is effective in reducing pain, improving quality of life and function, and decreasing opioids intake [20, 21]. As the population ages, the number of people with chronic pain will increase, leading to higher demand for physiotherapy services [22]. To ensure that the physiotherapy services offered to these people efficiently answer their needs, it is therefore important to understand and explore the scope of the perceived needs of people living with chronic pain. However, to our knowledge, no review has synthetised the extent of the literature on the perceived needs of this population. To date, reviews on the perceived needs in the physiotherapy field have mainly looked at the perceived needs of children with various conditions [23], of people living with non-specific low back pain [24] and of those having osteoporosis [25]. According to the results of these scoping reviews, patients expressed needs related to individually tailored interventions, exercises prescription, appropriate information and education, follow-up sessions and faster access to physiotherapy services. Furthermore, in a 2016 systematic scoping review, Wluka and colleagues explored the perceived needs of patients having one of five musculoskeletal conditions: inflammatory arthritis, osteoarthritis, back pain, neck pain and osteoporosis. The aim of their review was to understand patients’ perceived needs in regards of health information, health services and other non-medical services. Although this review did not solely look at physiotherapy services or chronic pain conditions, their results demonstrate that patients seek health information regarding the role of physiotherapy and its effects on body structures, as well as physiotherapy services that are individualized, easy to access and that include strengthening exercises [26].

The absence of a review on the perceived needs of people having chronic pain in regards of physiotherapy services is concerning since these people present distinct needs and report more unmet needs related to healthcare services than those without chronic pain [27]. For example, people living with chronic pain often don’t feel well understood by their healthcare professionals, including physiotherapists [28], which negatively affects their wellbeing and the healthcare services obtained [29]. Moreover, according to recent evidence, there are unmet needs regarding access to physiotherapy services for people living with chronic pain in Canada and other countries [30–32]. The demand exceeding the supply of physiotherapy services causes significant wait times to consult a physiotherapist [33–35], particularly affecting people living with chronic pain who are often disadvantaged by waiting list prioritization strategies [36, 37]. Furthermore, according to a 2019 report on access to physiotherapy services for people living with chronic pain in Canada, up to 45% of the respondents mentioned they would consult or consult more in physiotherapy if sessions were less expensive [38].

Thereby, our objective is to explore what is known from the existing literature about the perceived needs of people living with chronic pain regarding physiotherapy services.

## Methods

To meet our objective, we will conduct a scoping review. This type of review will allow us to describe and explore the extent of the literature on the needs of people living with chronic pain towards physiotherapy services and to identify gaps in the literature. This scoping review protocol was developed in accordance with Preferred Reporting Items for Systematic Reviews and Meta-Analyses PRISMA-Scr guidelines [39]. It follows the framework developed by Arksey and O’Malley [40] and enhanced by Levac et al. [41], Daudt et al. [42] and Peters et al. [43]. This scoping review protocol was registered with Open Science Framework (registration DOI: 10.17605/OSF.IO/6D8P3). This scoping review will generate new knowledge from published and publicly available studies. Therefore, no ethical approval is required to conduct this review.

Arksey and O’Malley’s framework describes six stages for conducting scoping reviews: 1) Identifying the research question; 2) Identifying the relevant studies; 3) Study selection; 4) Charting the data; 5) Collating, summarising, and reporting the results; 6) Consultation with stakeholders. Each of these stages will be detailed below.

This review will be conducted by a team of two students (one doctoral and one master’s student), supervised by two researchers having main expertise in physiotherapy services, and assisted by two other researchers, one with specific pain expertise and one with expertise in the organisation of primary healthcare. Two persons living with chronic pain who experienced physiotherapy services in the past are also part of the research team. All members of this team have contributed to the design of this review and will be involved in all upcoming key steps of the review.

### Stage 1: Identifying the research questions

#### Primary question

What are the perceived needs of people living with chronic pain regarding physiotherapy services?

#### Secondary questions

- What is the nature and extent of the current literature on this topic?
- Where are the gaps in the literature on this topic?
- How can perceived needs for physiotherapy services be described in terms of beliefs, values, preferences and expectations?

### Stage 2: Identifying relevant studies

The search strategy was developed in consultation with two librarians from two different universities. It was adapted for each of the targeted databases: Medline, Embase, CINHAL, APA PsycINFO (see S1 Appendix for details). No published filters will be used. The grey literature will also be searched using Google Scholar, OpenGrey [44] and ProQuest Dissertation & Theses Global (PQDTGlobal) [45]. A hand search of the reference lists of all selected documents will also be performed to find other relevant references. Abstracts from conferences will be excluded due to their lack of content describing the patient’s beliefs, values, preferences and expectations related to physiotherapy services. Systematic and scoping reviews will be excluded, but their reference lists will be examined to ensure relevant studies are included.

All studies that describe the perceived needs of adults (18 years old or older) with chronic pain regarding physiotherapy services, regardless of their methodology (i.e., quantitative, qualitative or mixed studies) will be included. All studies published in English and French will be considered regardless of their date of publication.

### Definition and key terms

*Patient*. “Patient” will be used to define any adult who have benefited or who could benefit from physiotherapy services. We will also include people who wanted to consult in physiotherapy but who were unable to do so due to accessibility reasons.

*Perceived needs*. As mentioned previously, the term “needs” is currently not well defined in the literature [7–9]. However, patients’ perceived needs can be associated with patients’ expectations and preferences [25]. Thereby, this review will consider perceived needs as related to patients’ beliefs, values, preferences and expectations regarding physiotherapy.

*Chronic pain*. Based on the 11^th^ edition of the International Classification of Diseases (ICD-11), chronic pain is defined as “persistent or recurrent pain lasting longer than 3 months” [1, 46]. It encounters chronic primary pain, chronic cancer pain, chronic postsurgical and posttraumatic pain, chronic neuropathic pain, chronic headache and orofacial pain, chronic visceral pain and chronic musculoskeletal pain [1]. Therefore, all types of chronic pain will be included in this review. This broad inclusion of the many existing types of chronic pain, regardless of the specific pathology involved or the origin of the pain will allow for the identification of a large variety of needs, from a large diversity of individuals living with chronic pain. More precisely, from the viewpoint of physiotherapy services, this choice will allow identifying the diversity of perceived needs of this group as a whole, regardless of each person’s condition. Indeed, it is possible to presume that the needs of people living with chronic pain are mainly influenced by the presence of chronic pain in itself, including its numerous consequences and impacts for individuals rather than by the etiology or the specific type of pain.

*Physiotherapy services*. All services provided by physiotherapists and technicians in physiotherapy, in any health care settings (e.g., publicly or privately funded services; rehabilitation centres, community health centre, home care rehabilitation, etc.), will be included. Tools, technologies and any other devices or applications such as YouTube or wearable technologies will be included as long as they are used and delivered by physiotherapists in order to provide exercises or advice or to support patients in their rehabilitation. Studies that include other healthcare services in addition to physiotherapy will be included if patients’ perceived needs, beliefs, values, preferences and expectations regarding physiotherapy are specifically described in the studies. However, studies describing patients’ needs related to multiple healthcare services without reporting results specific to physiotherapy services will be excluded. Studies providing results related to the outcome of a physiotherapy intervention without any mention of the expectations, preferences and lived experience of the patients will also be excluded.

### Stage 3: Study selection

Following the search, all initially identified articles and sources will be uploaded into Covidence (https://www.covidence.org/home) to first remove all duplicates. The first stage of screening will be based on titles and abstracts. Two independent reviewers will first examine a random sample of 50 references to assess the agreement between reviewers and to ensure that the eligibility criteria are relevant and clearly defined. Following this pilot screening and necessary adjustments, the same two persons will review all remaining titles and abstracts. Reasons for exclusion will be recorded. Any discrepancies between the two reviewers will be resolved through discussion between them. If required, a third reviewer will be consulted. Studies deemed potentially eligible following the first stage of screening will enter the second stage, which will be based on full-text examination. Before they pursue with this second stage, the two independent reviewers will meet and discuss about the first stage of screening to make any needed clarifications to the eligibility criteria. Again, disagreements will be resolved through discussions and, if required, by a third reviewer. The results of the selection process will be presented in a PRISMA-ScR flow diagram.

A pilot literature search was performed on April 27, 2022 on the four databases listed above to evaluate the number of studies that might be included in the review. Following the database search using our predetermined keywords, 4499 studies were imported into Covidence for screening. Once the duplicates removed, the first and second author screened the titles and abstracts to get an overview of the type and number of studies to be included. 294 studies were selected to be then reviewed as full text. This pilot search confirmed that several pertinent articles could be identified by our search and that we would obtain an appropriate number of studies to screen and include in our review.

### Stage 4: Charting the data

Covidence will be used for data extraction. A data extraction form will be used to extract data from the included studies and it will be uploaded into Covidence. Extracted data will include each study characteristics (e.g. author, study design, methodology), population (e.g. age, gender, localization of pain…) and information related to previous physiotherapy needs, beliefs, values, preferences and expectations.

The extraction form will be tested by two independent reviewers on five articles to ensure the feasibility and quality of the extraction process and to assess reviewers’ agreement. In case of disparities between the reviewers, they will meet to discuss and ensure concordance. If needed, the form will be modified and all changes will be mentioned in the publication of the scoping review.

Following this test, the same two reviewers will extract data from the remaining included articles. Any disagreements between the reviewers will be addressed through discussion or by consulting the other members of the team.

### Stage 5: Collating, summarising and reporting the results

Data from quantitative, qualitative and mixed methods studies will be analyzed and synthesised using convergent qualitative meta-integration [47]. Frantzen and Fetters defined convergent qualitative meta-integration as the process by which quantitative findings are transformed into qualitative data, then analyzed using qualitative methods, such as thematic analysis, and finally integrated with the findings from qualitative studies. Data arising from quantitative studies are thereby “converted into qualitative themes by utilizing descriptive conclusions from the quantitative papers without using the numeric results” [48].

Frantzen and Fetters proposed seven steps for convergent qualitative meta-integration [47]. Once the literature search is done (first step) and the included studies are categorised as quantitative or qualitative studies (second step), quantitative data needs to be transformed into qualitative data (third step). After this transformation, an iterative intra-method analysis and synthesis is performed (i.e., quantitative studies are analyzed together, as are qualitative studies–fourth step) followed by an iterative inter-method analysis and synthesis (i.e., findings from the analysis and synthesis of the quantitative and qualitative studies are brought together–fifth step). Finally, results are organised and compared (sixth step) and conclusions are draws (seventh step). If mixed-methods studies are included, another step will be added to the meta-integration process. This eight step called fractionation will be performed before the transformation of the quantitative data and will be used to separate the quantitative and qualitative data of the mixed-methods studies [47]. Throughout the data transformation step, the intra-method analysis step and the inter-method analysis steps, inductive thematic analysis will be used to determine the major themes described in the different studies [43]. One reviewer will perform this analysis using the Nvivo software. By the end, central themes will be summarized, and findings will be presented for each of the objectives of this scoping review. The results of this analysis will then be presented and discussed with all members of the team, including two persons living with chronic pain (see Stage 6: Consultation with stakeholders). A visual representation of the findings will be created.

Considering the subjective and qualitative nature of the perceived needs, which is the subject of interest of this scoping review, convergent qualitative meta-integration will allow a deeper understanding of this complex concept by efficiently combining the results of qualitative, quantitative and mixed studies [47]. Through this analysis process we will be able to identify what are the perceived needs of people living with chronic pain regarding physiotherapy services and to identify if gaps exist in the current literature related to this topic.

Quality assessment of the included studies will not be performed during this scoping review. As our purpose is to map the available literature on the perceived needs of people living with chronic pain in order to identify those needs, identify the gaps in the existing literature, and understand how those needs can be described in terms of beliefs, values, preferences and expectations, we prefer to include and analyze the full scope of studies, regardless of their quality [43].

### Stage 6: Consultation with stakeholders

Two persons living with chronic pain and who previously used physiotherapy services have been involved in review preparation as regular members of the research team and will continue throughout the research process. More specifically, they have already been part of the development of the research protocol and the definition of the research question and objectives (Stage 1: Identifying the research questions). They will also be actively engaged in the discussions surrounding the analysis process using convergent qualitative meta-integration (Stage 5: Collating, summarising and reporting the results). These persons were recruited from two different associations of persons living with chronic pain in the province of Quebec, Canada. Their collaboration will help strengthen the interpretation of results because of their personal experiences with pain and physiotherapy. Since both persons are also involved in associations for people living with chronic pain, they will also be well placed to facilitate knowledge transfer to this population.

## Discussion

This review will provide an overview of the existing literature about the perceived needs of people living with chronic pain regarding physiotherapy services. It may also bring valuable information to better define the concept of perceived needs and how patients expectations and experiences influence those needs.

In terms of potential impact, this scoping review will raise awareness about the diversity and complexity of the needs perceived by people living with chronic pain regarding physiotherapy. Although a patient-centered approach meeting patients’ needs is highly recommended for the management of people living with chronic pain, needs evaluated by healthcare services representatives have been better documented and used to organise healthcare services than the needs perceived by the patients themselves. Thus, the identified perceived needs in this review may inform stakeholders on needs to consider to truly answer patients’ needs and thus, to offer more ethical and higher quality physiotherapy services.

Finally, results of this review will inform a mixed-methods study looking at how the current clinical practices and organisational politics of physiotherapy services concretely respond to patients’ perceived needs.

## Supporting information

S1 Checklist PRISMA-P (Preferred Reporting Items for Systematic review and Meta-Analysis Protocols) 2015 checklist: Recommended items to address in a systematic review protocol*. (DOC)

S1 Appendix Search strategy applied to Medline, PsycInfo, Embase and CINHAL. (DOCX)
